# Inhalation of Sulphuric Ether

**Published:** 1847-06

**Authors:** 


4. *Inhalation of Sulphuric Ether.*—Our exchange Journals continue to be crowded with cases and discussions illustrative of the effects of this kind of medication. Not merely is it employed when cutting instruments are used, but in the treatment of luxations, hernia, and labor, especially in instrumental cases; in asthma, croup, and nearly all the ills that flesh is heir to. These numerous experiments will doubtless lead to a better understanding of its powers and adaptations, and it will be strange if much good does not result. Bad consequences are known to have followed the employment of the remedy in repeated instances but the only wonder is, with such indiscriminate use of so powerful an agent, that the number of accidents has not been greater. That our readers may know something of what is said and doing on the subject, at home and abroad, we have, at the sacrifice of variety, devoted a considerable portion of the present number to extracts bearing on the most important points involved in the discussion. Already, in this country, we think we discover a decline of the excitement in relation to it, which prevailed so extensively at the first.—*Med. Exam.*

